# Retrospective pig welfare assessment at rendering plants – a useful tool for monitoring and inspection

**DOI:** 10.1186/s40813-026-00504-6

**Published:** 2026-04-28

**Authors:** Elisabeth grosse Beilage, Kristina Maschat, Johannes Baumgartner

**Affiliations:** 1 Field Station for Epidemiology, University of Veterinary Medicine Hannover, Foundation, Büscheler Str. 9, 49456 Bakum, Germany; 2 Centre for Animal Nutrition and Welfare, Clinical Department Farm Animals and Food System Science, University of Veterinary Medicine Vienna, Veterinärplatz 1, Vienna, 1210 Austria

**Keywords:** Fallen stock, External examination, Pathology, Pain, Suffering, Euthanasia

## Abstract

**Background:**

The COUNCIL DIRECTIVE 98/58/EC (1) states that animal owners and care takers must ensure that animals under their care are not subjected to unnecessary pain, suffering and injury. Until now, rendering plants as a potential ‘exit’ in animal production have been largely ignored as a potential assessment site for animal welfare monitoring. The aim of the present work was to provide an overview of welfare-related lesions commonly found in pigs at rendering plants. Data collection was carried out in Austrian and German rendering plants and focussed on lesions that were expected to be recognizable for any pig farmer.

**Results:**

In the Austrian study, 20.8% of all pigs delivered to a rendering plant showed lesions potentially associated with substantial pain and/or suffering. In Germany, 16.0% of examined pigs (sample A) may have been exposed to substantial pain and/or suffering. Most common lesions were biting wounds, ulcerative skin lesions and swellings.

**Conclusions:**

Based on the results it must be assumed that a considerable number of pigs experienced substantial pain and/or suffering prior to death. External examinations focusing on potentially welfare-relevant lesions carried out at rendering plants could be helpful in identifying farms at high risk of non-compliance with the Animal Welfare Act.

## Background

According to COUNCIL DIRECTIVE 98/58/EC [1] Member States must ensure that owners and/or care takers initiate all reasonable steps to ensure the welfare of animals under their care and to prevent them from experiencing unnecessary pain, suffering or injury. This includes the obligation to identify diseased and injured animals at an early stage, to handle them appropriately, and to provide adequate veterinary treatment. Authorized rendering plants are entrusted with the collection, […] processing, storage, […] or disposal of animal by-products which includes carcasses from fallen stock [2].

Official inspections of pig herds have shown that deficiencies in the management of diseased and injured pigs lead to considerable welfare problems. Inadequate care and treatment of diseased and injured animals, and lack of timely killing of pigs when they are unlikely to recover, are a major part of the problem [3]. According to reports from official veterinarians working in animal rendering plants, it is not uncommon for carcasses to be delivered with signs of suffering. In these cases, it can be assumed that owners or care takers have failed in their duty of care [4].

Traditional, random on-farm animal welfare inspections may not always be helpful in promptly identifying farms at high risk of failing to provide adequate care for diseased and injured animals [5]. Rendering plants, like slaughterhouses, function as essential terminal nodes in the swine production chain, forming logistical bottlenecks through which all pigs must pass at the end of life. In future, suspected cases of animal neglect could theoretically be efficiently identified at this stage, enabling prompt corrective measures and legal action, with inspection outcomes integrated into a risk-based animal welfare control system. However, the routine process of carcass disposal does not currently include any form of animal welfare assessment [5, 6].

The first aim of the studies, carried out in Austria and Germany was to provide an overview of the type and severity of welfare-related lesions commonly found during external inspection of pigs delivered to rendering plants. Emphasis was placed on lesions a pig farmer would be expected to recognise. A further aim was to describe findings most likely to be associated with substantial pain and/or suffering [7–9], which would clearly indicate that timely killing had not been carried out.

## Methods

For this survey, findings related to substantial pain and suffering were summarised from two studies conducted at rendering plants in Austria [10] and Germany [11]. The Austrian study was conducted over a total of 20 days during the summer at a single rendering plant located in Lower Austria and included 978 pigs from 100 truckloads. In the German study, 632 pigs from 57 truckloads, were examined to estimate the prevalence of pigs with findings related to substantial pain and suffering (sample A). For the detailed description of the variation of findings indicative of pain and suffering, 101 pigs selected from sample A and another 362 pigs conveniently selected from more than 100 additional truckloads were examined (sample B). In these 100 + truckloads, so many pigs were delivered that space constraints at the rendering plants forced the examiner to focus on pigs in the top layer of the load with obvious signs of welfare related findings. Therefore, sample B consisted of preselected pigs with animal welfare related findings associated with pain and suffering (Table 1). Due to the targeted selection of suspect pigs, the prevalence of lesions in sample B is not representative of the average of pigs delivered to rendering plants. The German study was carried out in 19 days (winter/spring) across in four rendering plants located in different parts of the country. The areas from which the pigs were supplied are located in 6 federal states and are characterised by high pig density and medium to large pig herds, as well as lower pig density and very large pig herds or low pig density and small pig herds. Both studies included fattening and breeding pigs while the examination of piglets was restricted to those with an estimated body weight of more than 10 kg (German study) or 25 kg (Austrian study).

**Table 1:**
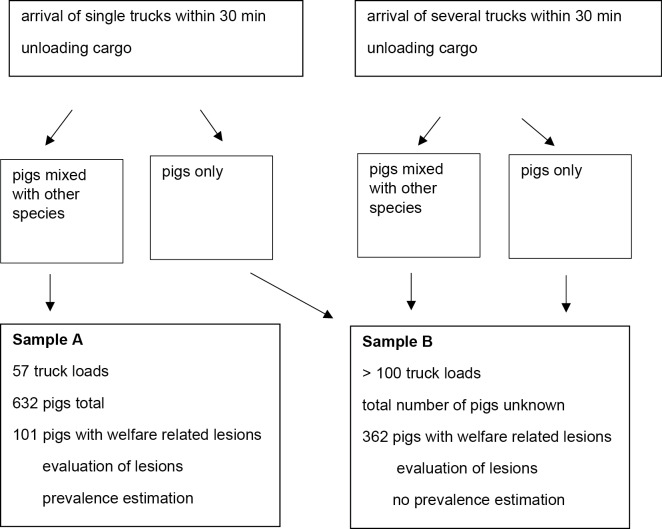
Sampling scheme in the German study

The Austrian study aimed to provide an initial overview on lesions in pig carcasses at rendering plants potentially related to animal welfare issues [10]. For this purpose, all pig carcasses unloaded from a truck were examined and prior cleaning was limited to the claw region. Individual cases, representative of specific lesions, were photographed. In the German study, pigs suspected of having welfare related findings were identified after the trucks were unloaded at the rendering plant. First, each selected pig was then photographed in the condition in which it was delivered. Photographs were taken from both sides, first in overview (lateral position, frontal view from above and lateral position with diagonal view from the front) followed by details of the lesions. The lateral position with diagonal view from the front is useful for assessing the body condition as prominent bones are more visible due to the shadow cast. In the next step, all selected pigs were cleaned with water and the photographic documentation was repeated. In both studies, all lesions were recorded on a handwritten sheet. Finally, the lesions were scored, based on the photographs and handwritten records and transferred to an Excel file (Table 2).

**Table 2:** Scoring of findings and lesions on external examination of dead pigs at rendering plants

Parameter	Score/ category	Criteria	Study Austria	Study Germany
Age category	piglet	weaned piglet (10–30 kg body weight)	≥ 25 kg	✓
	fattening pig	fattening pig (31–120 kg body weight)	✓	✓
	breeding pig	breeding sow, gilt, boar (> 120 kg body weight)	✓	✓
Body condition	1 mild weight loss	subcutaneous fat and muscles not in an optimum shape		✓
	2 moderate weight loss	visible to some extent: *Tuber spinae scapulae*, ribs, spine processes		✓
	3 emaciation/ syn. cachexia	clearly visible: spine of the scapula, ribs, spinous processes of the vertebra, iliac bone scoops or ischial tuberosities of the pelvis, distinct concave line in the gluteal region	✓	✓
Swelling	1	one or more clearly visible enlargement(s)	✓	assessed as enlargement(s) of joint(s) only (shoulder, elbow, carpus, hip, knee, tarsus)
Foot	1	clearly visible in claw/dewclaw: overgrowth, deformation, torn horn shoe, heel lesions, deep coronary band lesions	✓	✓
Skin	1	ulcerative lesion: mild, superficial, with reddened but intact skin	✓	✓
	2	ulcerative lesion: deep with perforated skin, underlying tissue not exposed	✓	✓
	3	ulcerative lesion: all skin layers and underlying tissue affected	✓	✓
	moderate	ulcerative lesion < 5 cm diameter	✓	
	large	ulcerative lesion ≥ 5 cm diameter	✓	
Biting lesion	1	skin perforated, small bleedings, mild inflammation	✓	limited to tail and ear lesions
	2	loss of substance, necrosis, moderate to severe inflammation		limited to tail and ear lesions
Navel	1	Umbilical outpouching with ulcerative, deep skin lesion (see grading ulcerative skin lesions)		✓
Rectum	1	Abdomen: extensive distension		✓

*For all parameters, the normal finding was scored ‘0”

In the Austrian study, all findings other than score ‘0” were considered potentially associated with substantial pain and/or suffering, whereas in the German study mild weight loss (body condition score 1), mild superficial skin lesions (skin score 1) and biting lesion with small bleedings or mild inflammation (biting lesion score 1) were considered associated with welfare impairment but not with substantial pain and/or suffering.

**Fig. 1:**
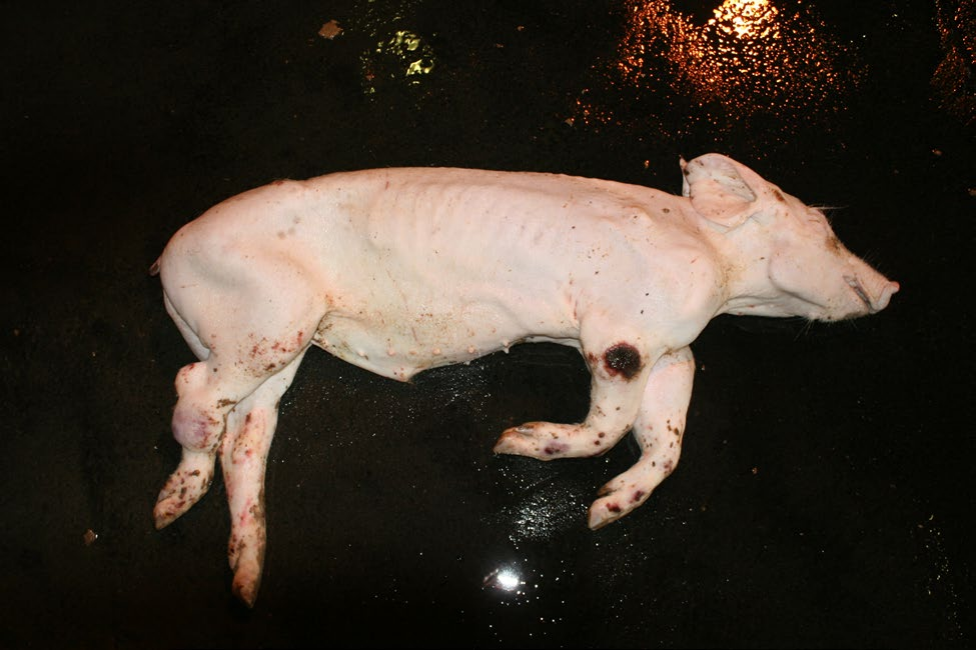
Emaciation with ribs and *tuber spinae scapulae* clearly visible

**Fig. 2:**
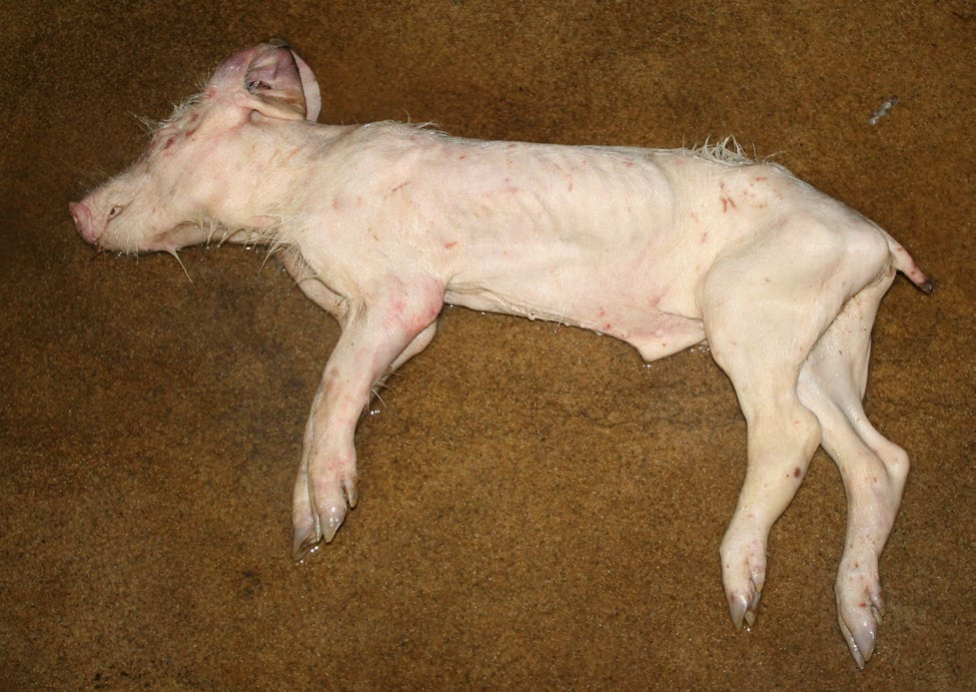
Emaciation with ribs and prominent bone points clearly visible, thigh muscles almost ‘straight” in caudal direction

The diagnosis of ‘emaciation’ was made when fat tissue and muscles had regressed to such an extent that the ribs and bone prominences such as the spinous processes, scapula or *tuber spinae scapulae*, pelvis were visible (Figs. 1 and 2). Muscle atrophy, particularly of the *M. longissimus dorsi* and the thigh muscles was also considered. In cases of severe emaciation, the femur and pelvis were clearly visible under the skin (Fig. 1). A subset of carcasses (*n* = 12) with moderate manifestation of the above criteria was necropsied. The diagnosis, previously made on the basis of the external examination findings, was confirmed in each of these animals by the atrophy of the coronary adipose tissue.

Data were organised in Microsoft Excel^®^ (Version 2010, Microsoft Corporation, Redmond, Washington, US) and transferred into the statistical analysis program in IBM SPSS Statistics Version 22 (IBM Corporation, 2013) for further evaluation (Chi square test, Fisher’s exact test). P values less than 0.05 were considered statistically significant.

## Results

### Prevalences of welfare related findings associated with substantial pain and suffering

The external examination of pig carcasses (*n* = 978, in 100 truckloads) delivered to a rendering plant in Austria revealed a prevalence of findings considered potentially associated with substantial pain and/or suffering in almost 21.0% of the animals.

In Germany, findings potentially indicative of substantial pain and/or suffering were found in 16.0% of the carcasses from sample A (Table 3). Sample B contained pre-selected carcasses and was, therefore, only used for the detailed description of the welfare-related findings (see below).

**Table 3:** Prevalences of animal welfare related findings considered associated with substantial pain and/or suffering in carcasses delivered to rendering plants in Austria or Germany

	Country		
	Austria^1^	Germany^2^	
		sample A*	sample B**
piglets	180	0	*137*
fattening pigs	641	485	*272*
breeding pigs	157	147	*54*
total	978	632	*463*
carcasses with welfare related findings associated to pain/suffering	203 **20.8%**	101 **16.0%**	*362*

^1^ (10), ^2^ (11); * sample A was used for prevalence estimation and included the evaluation of all pig carcasses delivered from 57 truckloads; ** sample B (*n* = 463) included the 101 pig carcasses with welfare-related findings from sample A and a further 362 pig carcasses from more than 100 additional truckloads where it was not possible to examine all pig carcasses delivered due to the high number of animals

In the following section, the most common findings and evaluation criteria are explicated separately for each lesion. In this section, only the number of cases/carcasses available to explain the findings is presented to avoid drawing conclusions from percentages that are based on a sample (sample B) that is not representative of the population.

### Emaciation (syn. cachexia)

**Fig. 3:**
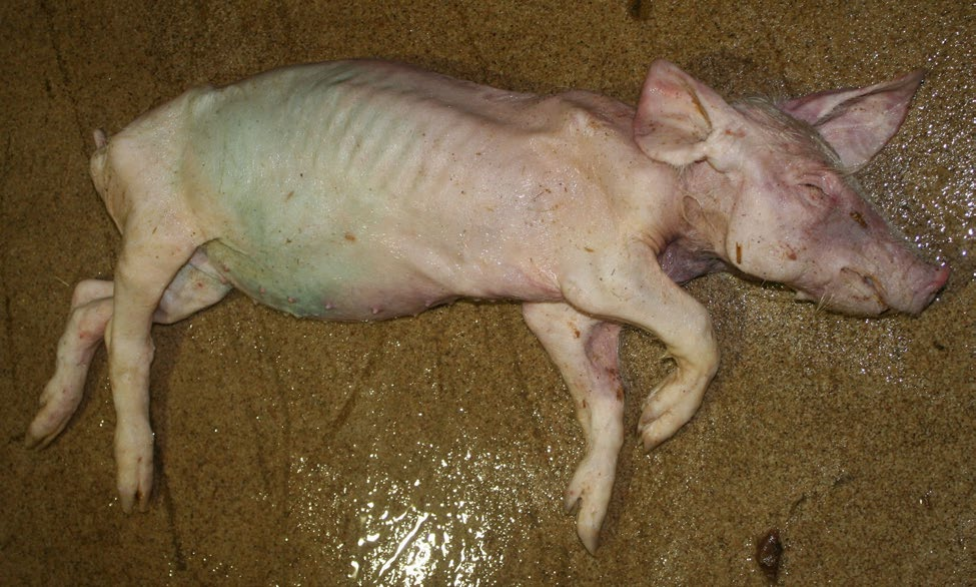
Piglet carcass with emaciation

**Fig. 4:**
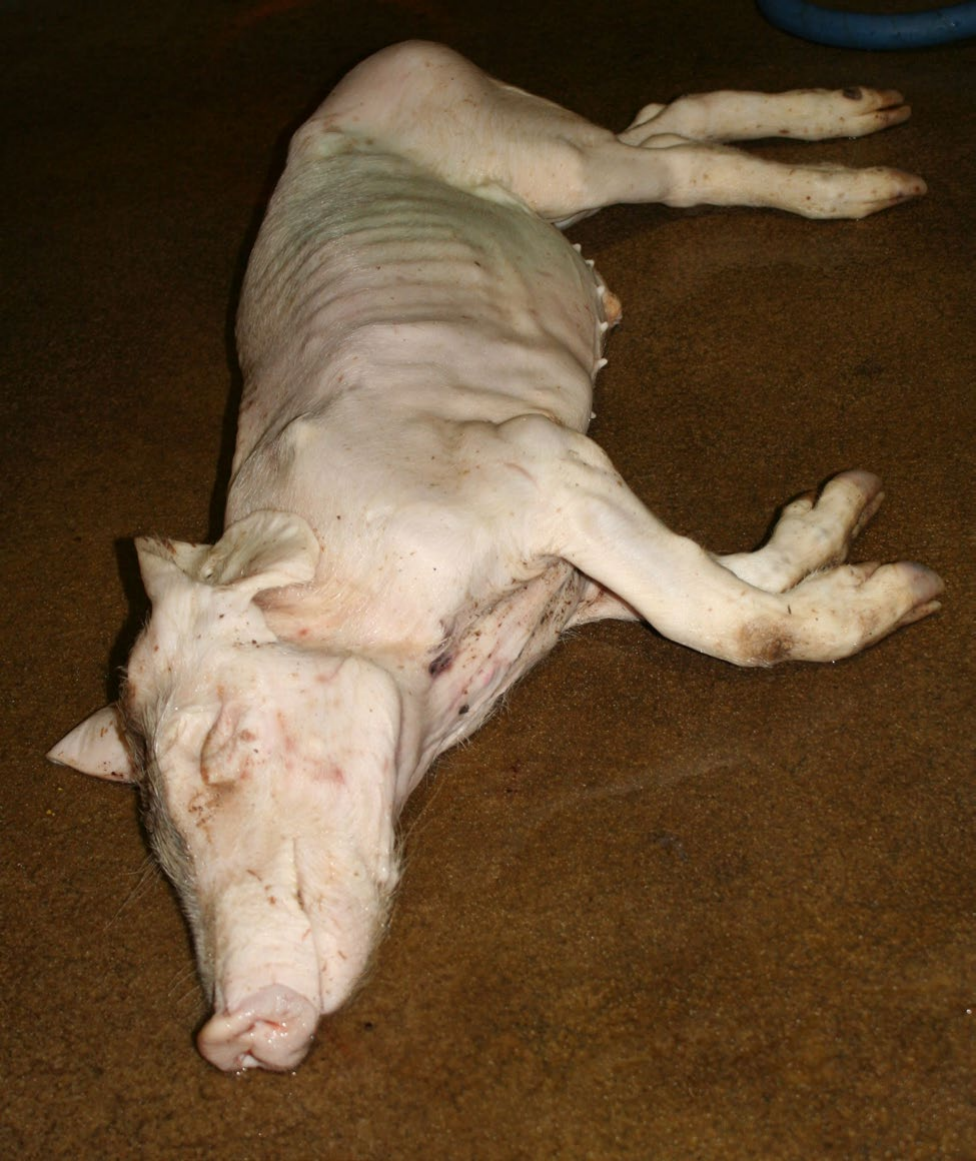
Fattening pig carcass with emaciation

Very poor body condition was assessed in 6 carcasses in the Austrian study and 215 carcasses in the German study. In these cases, the bones of the chest, the spinous processes of the vertebra and the iliac bone scoops and ischial tuberosities of the pelvis were clearly visible. Visibility of the vertebral and pelvic bones requires a significant reduction in fat and muscle mass (Figs. 3 and 4). In the German study, carcasses showing emaciation were significantly more likely to have long hair, ulcerative skin lesions, bite wounds on the tail and ears, and claw lesions than those in unimpaired body condition (Table 4).

**Table 4:** German study – Occurrence of other findings in carcasses with emaciation or mild to moderate weight loss compared with carcasses in unimpaired body condition

finding	body condition		
	Emaciation (*n*) (%)	mild to moderate weight loss (*n*) (%)	Unimpaired (*n*) (%)
hair, long	155 72.1% ^a^	71 44.3% ^b^	5 5.7% ^c^
skin, ulcerative lesion (superficial or deep)	94 43.7% ^a^	64 40.0% ^a, b^	25 28.5% ^b^
tail, bite lesion with loss of substance	112 52.1% ^a^	62 38.8% ^a, b^	25 28.4% ^b^
ear, bite lesion with loss of substance	62 28.8% ^a^	31 19.4% ^b^	8 9.1% ^b^
claw, dew claw or coronary band lesion	28 13.2% ^a^	37 23.1% ^b^	18 20.5% ^b^
joint enlargement	75 34.9% ^a^	68 42.5% ^a^	26 29.5% ^a^
Total	215	160	88

Different letters indicate significant differences (*p* < 0.05)

### Swellings

**Fig. 5:**
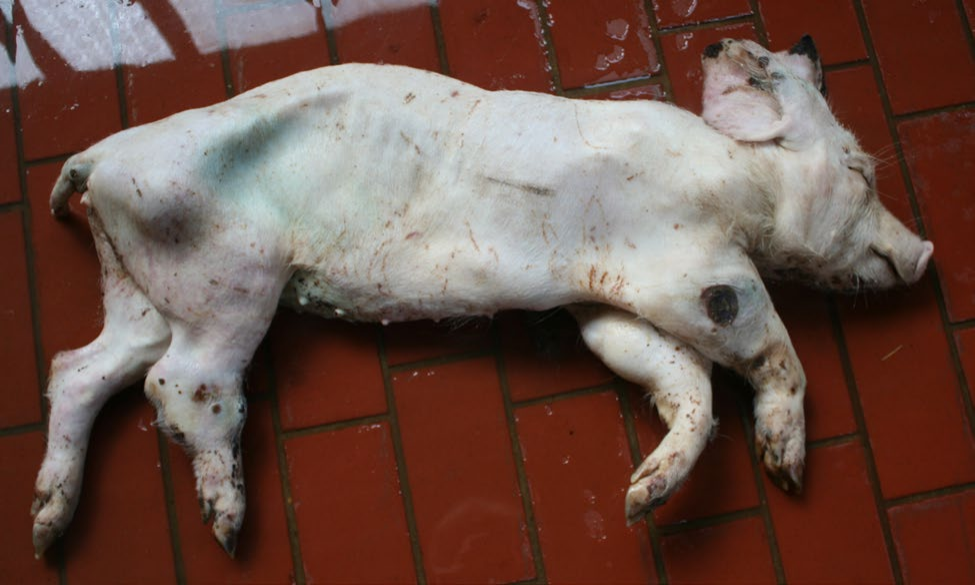
Piglet carcass with polyarthritis (elbow, tarsus) and ulcer, emaciation and multifocal inflammatory skin lesions

**Fig. 6:**
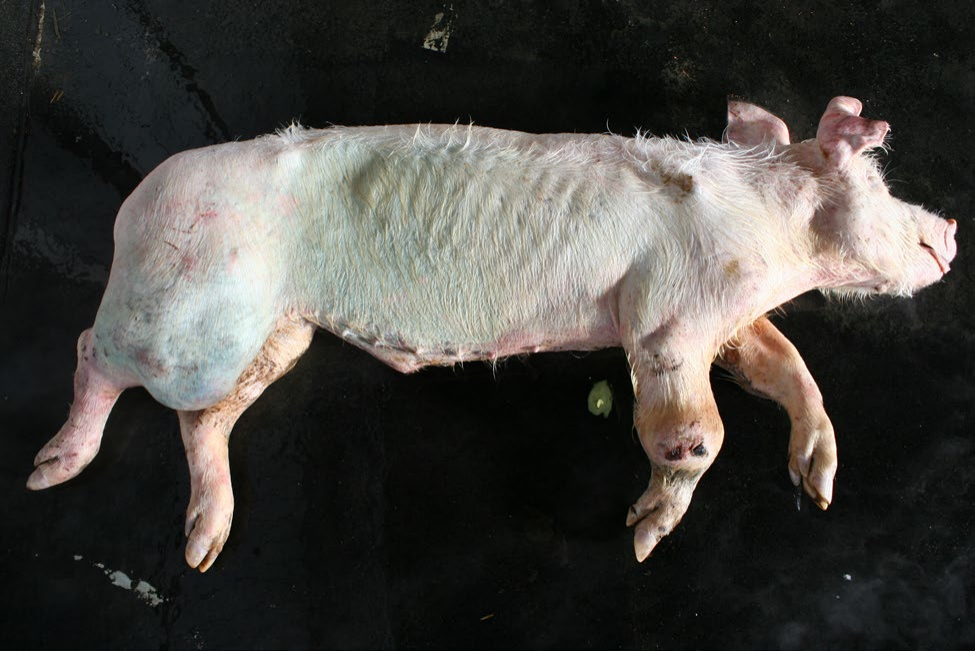
Fattening pig carcass with polyarthritis (carpal joint, knee), emaciation, decubitus and long hair

**Fig. 7:**
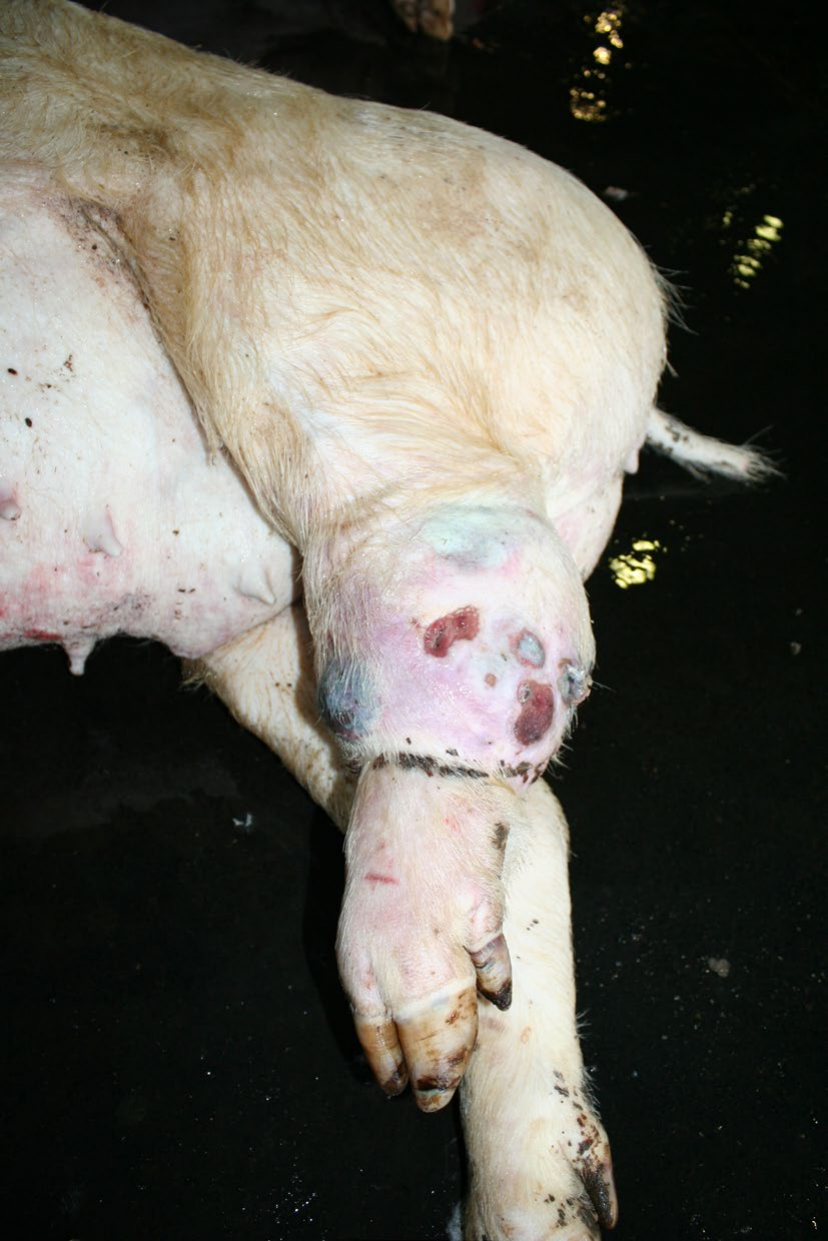
Sow carcass with arthritis (tarsus)

In the Austrian study, substantial enlargements, mainly in the joint area, were documented in 30 carcasses. In a subsample of 14 cases, incisions were made and a purulent alteration was determined in all lesions. In the German study, enlargements in the area of joints were found in 169 carcasses, 38 of which showing more than one joint affected (Figs. 5, 6 and 7). Incision of the enlargement in 19 carcasses revealed purulent arthritis in all lesions.

In the German study, 43 out of 169 carcasses exhibited pronounced joint enlargements in conjunction with decubital ulcers. Carcasses with substantial enlargements in the area of at least one joint were significantly more likely to have ulcerative skin lesions (161 out of 169 vs. 137 out of 294, *p* < 0.05) as well as tail biting lesions (109 out of 169 vs. 90 out of 294, *p* < 0.05) than carcasses without enlargement of the joints.

### Foot lesions

**Fig. 8:**
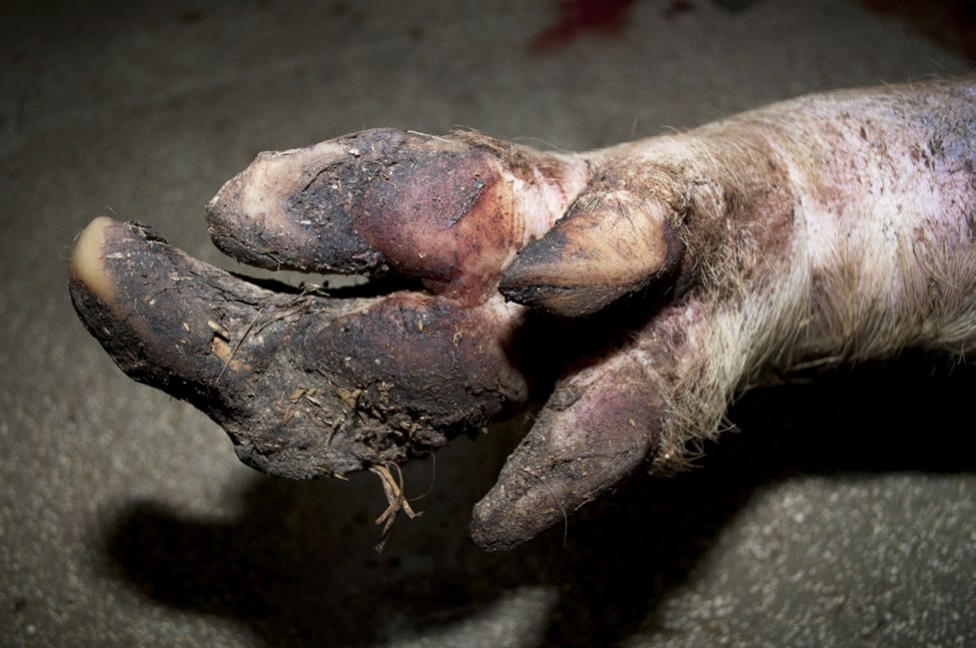
Breeding pig carcass with an overgrown and deformed lateral claw and overgrown dewclaws

**Fig. 9:**
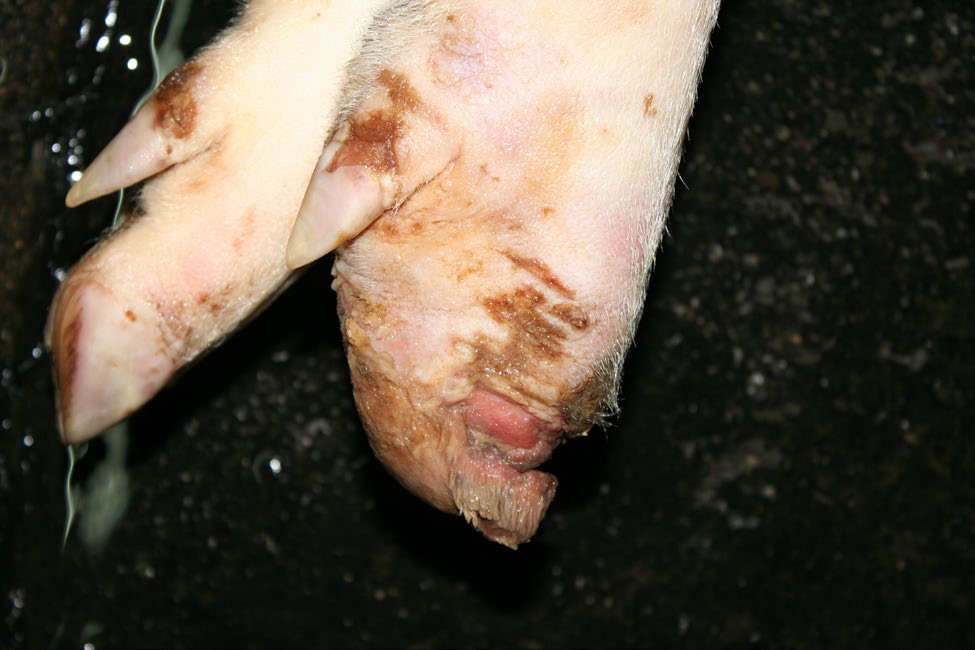
Fattening pig carcass with a torn horn shoe

**Fig. 10:**
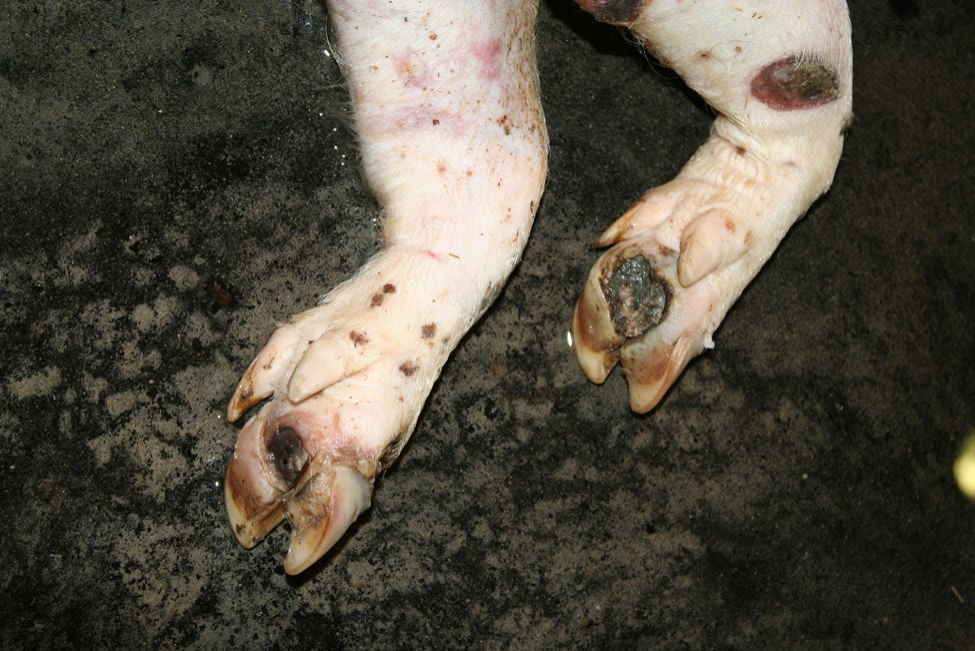
Fattening pig carcass with deep necrotic heel lesion and decubitus (lateral tarsus)

**Fig. 11:**
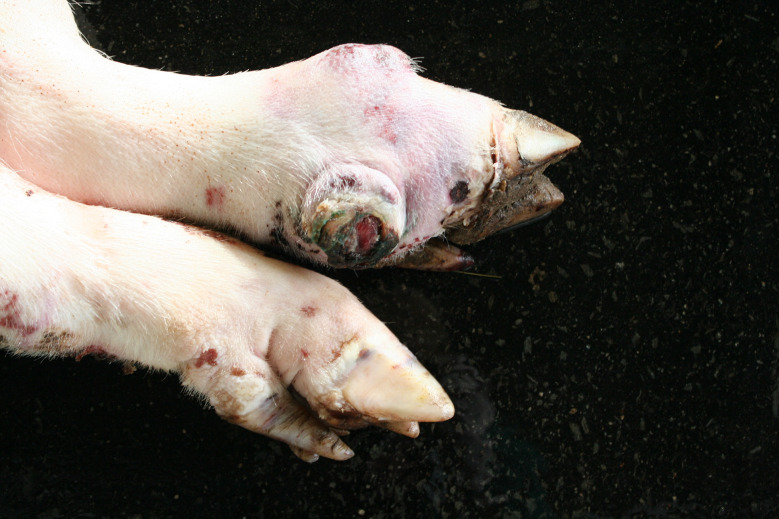
Sow carcass with complete tearing of the dew claw and inflammatory reaction of the surrounding tissue

**Fig. 12:**
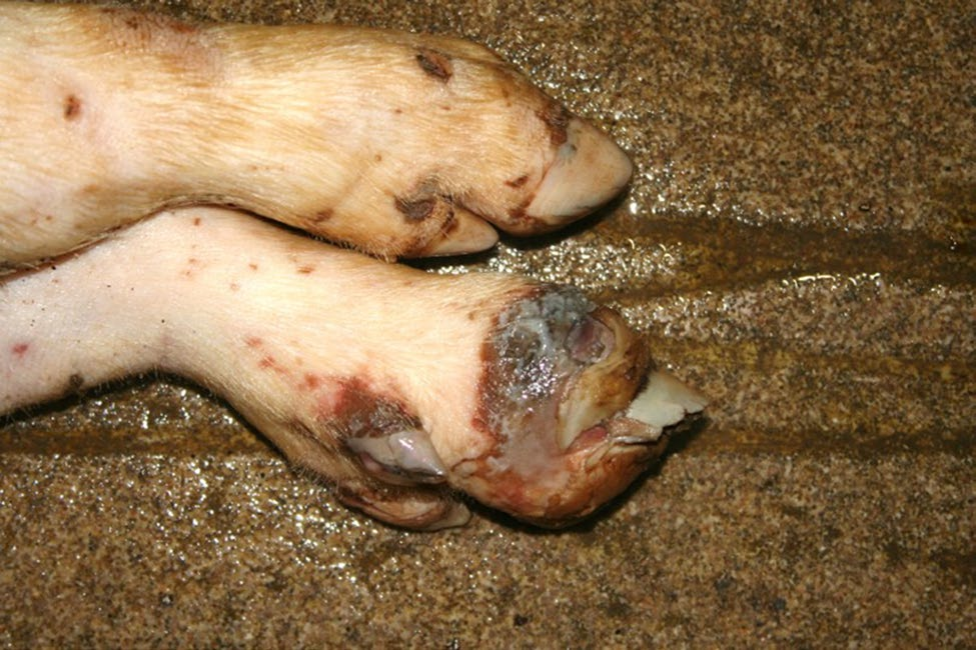
Fattening pig carcass with deep coronary band lesion

In the Austrian study, foot lesions were found in 23 carcasses of which 16 were breeding pigs. In the German study, foot lesions were found in a total of 60 carcasses. Of these, breeding pigs were most commonly affected (21 out of 54 breeding pigs). Foot lesions included claw and dewclaw alterations (overgrowth, torn or injured horn shoe) and heel lesions (Figs. 8, 9, 10 and 11). In addition, the German study identified 18 cases of deep coronary band lesions (‘bush foot’) (Fig. 12). Breeding pigs were more likely to be affected than younger pigs (8 out of 54 vs. 10 out of 409, *p* < 0.05).

### Ulcerative skin lesions

**Fig. 13:**
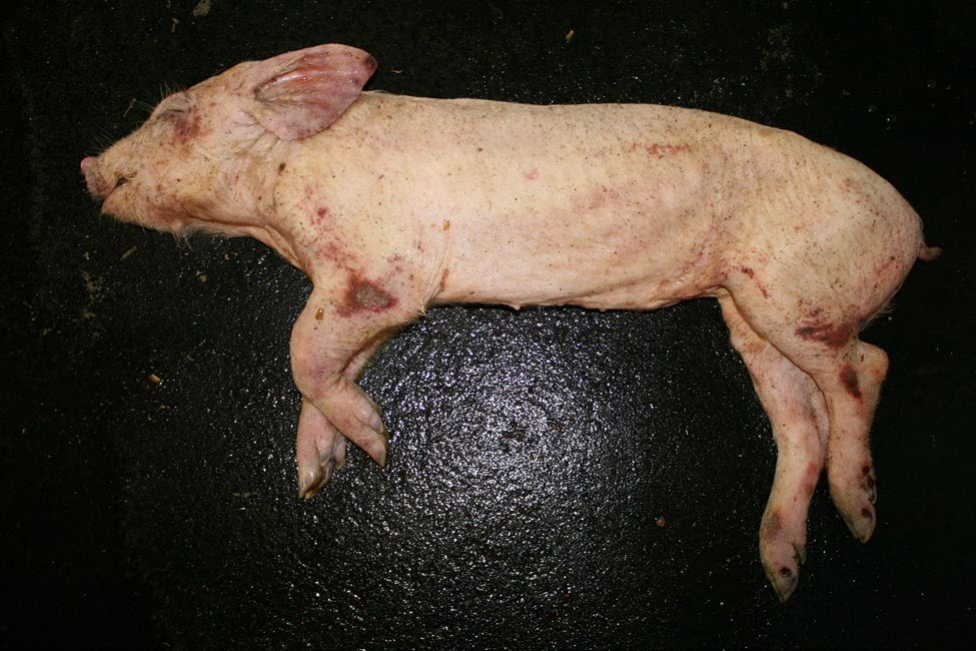
Piglet carcass with superficial ulcerative skin lesion

**Fig. 14:**
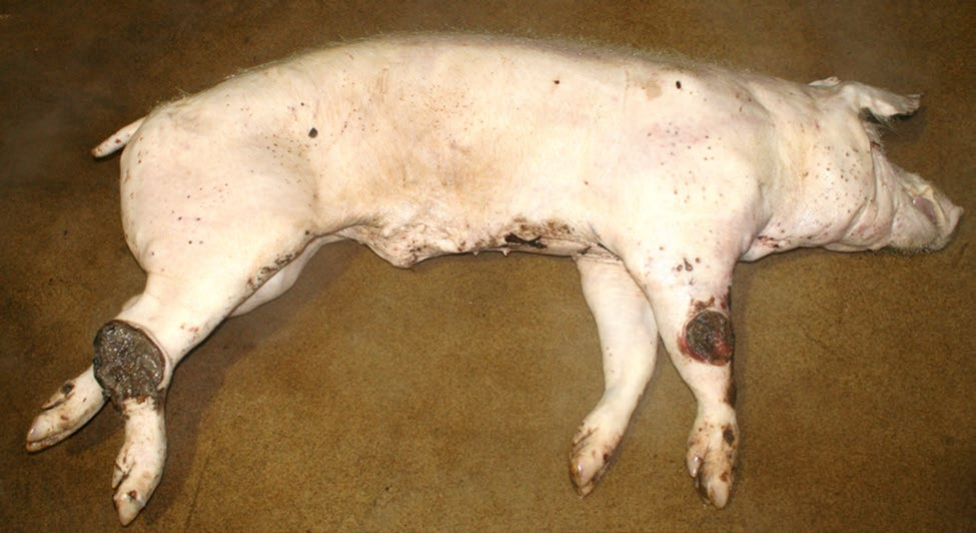
Fattening pig carcass with large perforating ulcerative skin lesion

In this paper, the category of ulcerative skin lesion summarises pressure sores and ulcers seen in various parts of the body. Superficial lesions (Fig. 13) were identified in 13 carcasses in the Austrian study. In the German study, superficial ulcerative lesions were found in 101 carcasses. Perforating ulcerative lesions affecting deeper skin layers were found in 67 carcasses examined in Austria and 82 carcasses examined in Germany. In the German study most ulcerative lesions were of moderate size (diameter < 5 cm) (Figs. 5 and 10). In some cases, however, ulcerative lesions were large (diameter ≥ 5 cm) and perforating, and were evaluated as a separate condition (Fig. 14).

### Biting lesions

**Fig. 15:**
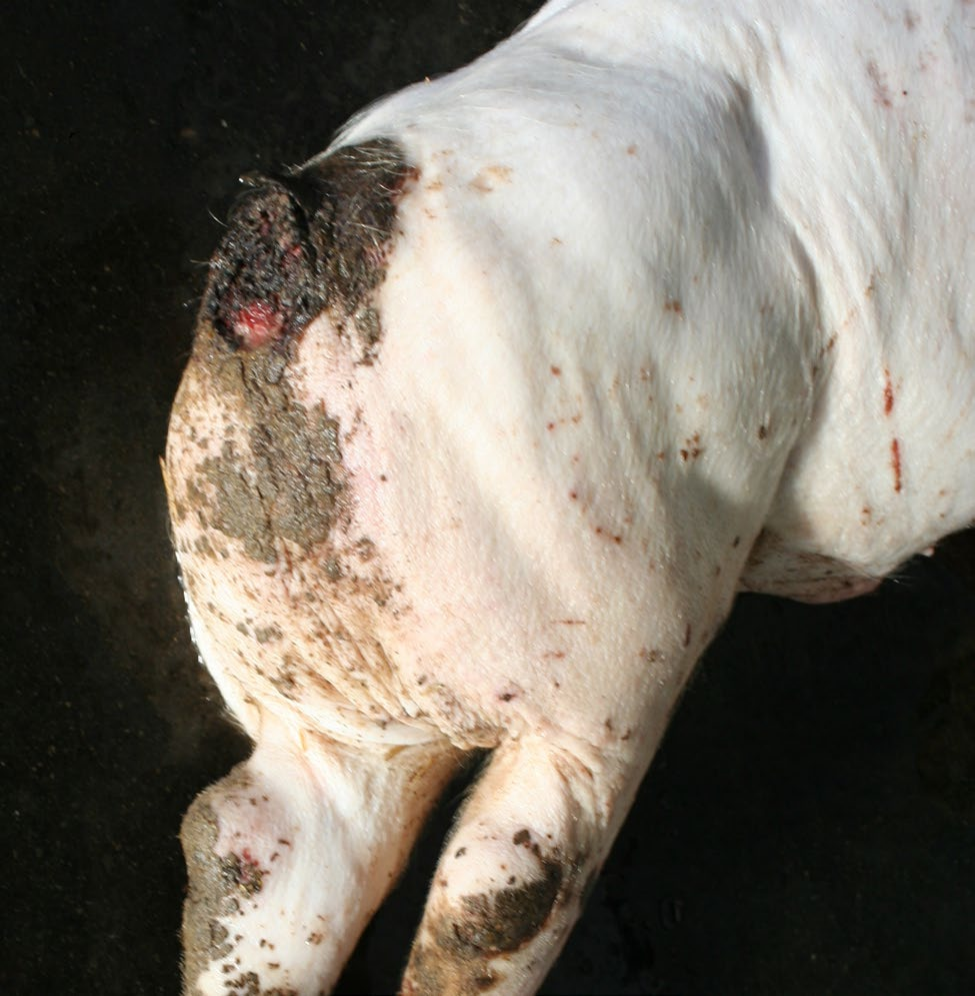
Fattening pig carcass with tail biting lesion extending to the tail root and surrounding tissues

The Austrian study summarised biting lesions on tails, ears and other body regions and reported 103 affected carcasses. In the German study, tail biting lesions were identified in 206 carcasses. In 131 of the 206 carcasses the tail biting lesions showed a combination of substance loss with signs of necrosis and inflammation (Fig. 15) while the other cases show only one or two of these lesions. In 14 carcasses only tail necrosis without signs of biting was observed. In 39 carcasses the tail was not assessed due to poor quality of the photographs and in 204 cases the tail was unaffected. Ear biting lesions were found in 118 carcasses, while 22 cases showed only necrosis without signs of biting. The ears were unaffected in 318 carcasses and in 5 cases the quality of the photographs was not appropriate for evaluation.

### Umbilical outpouchings

**Fig. 16:**
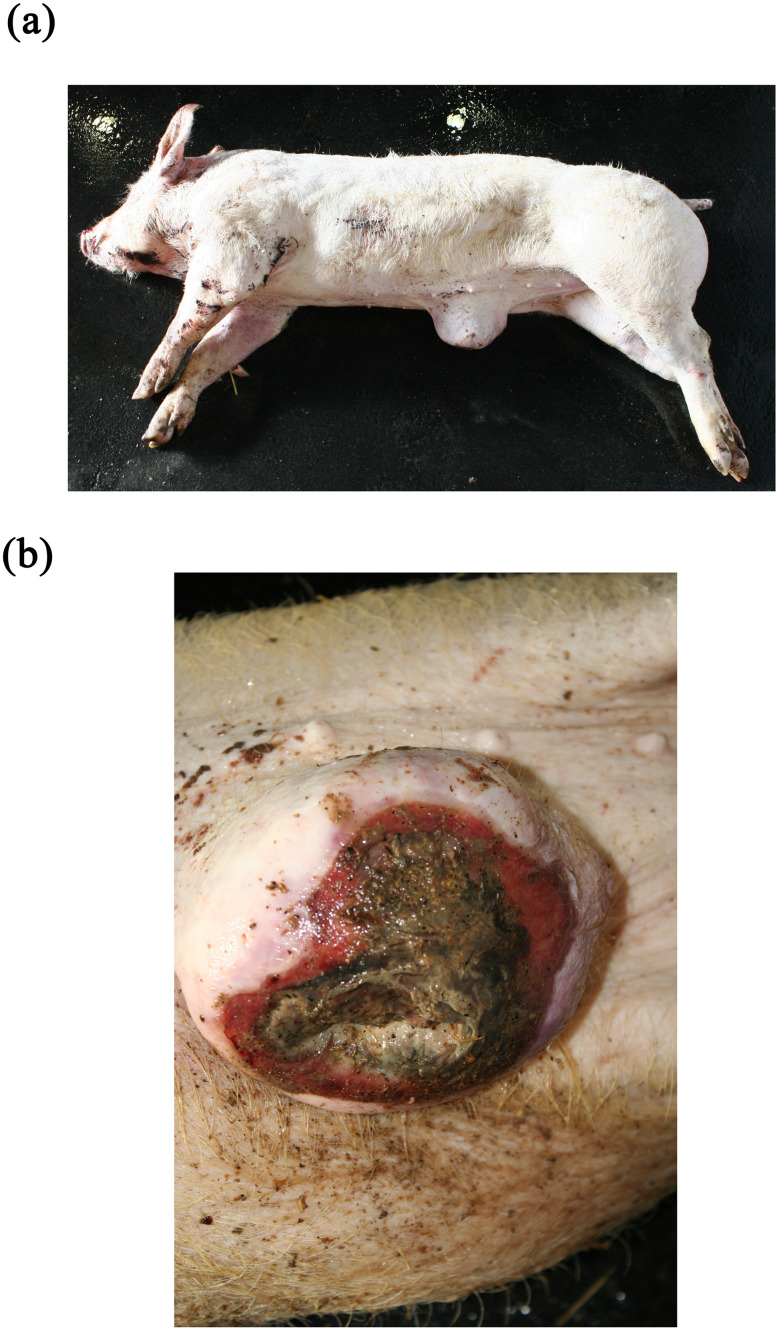
**a** Fattening pig carcass with a medium sized umbilical outpouching **b** Fattening pig carcass – same pig shown in **a** – with large and deep necrotic lesion of the umbilical outpouching

**Fig. 17:**
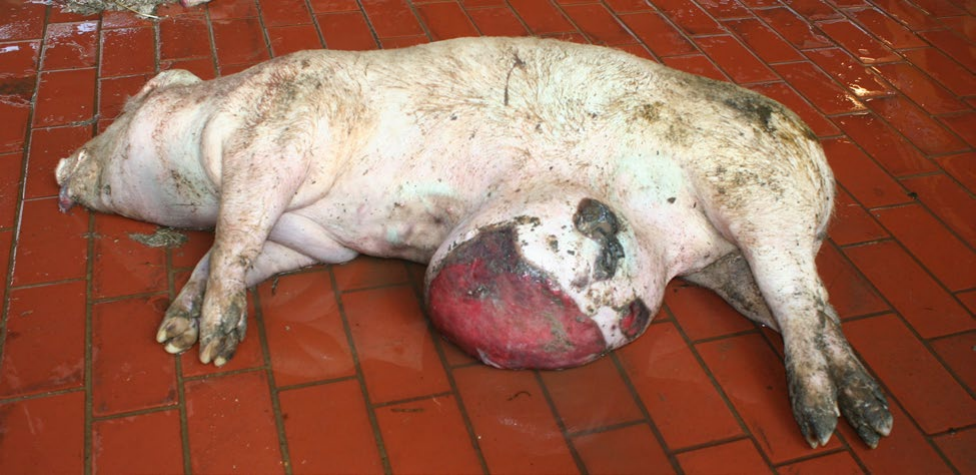
Fattening pig carcass with large and deep necrotic lesions of the umbilical outpouching

The evaluation of umbilical outpouchings was part of the German study but not included in the Austrian study. Large umbilical outpouchings with a diameter ≥ 20 cm were found in 28 carcasses. In 13 of the cases extensive and/or deep skin lesions were observed, developing from the ventral aspect of the outpouching (Figs. 16b and 17).

### Rectal stricture

**Fig. 18:**
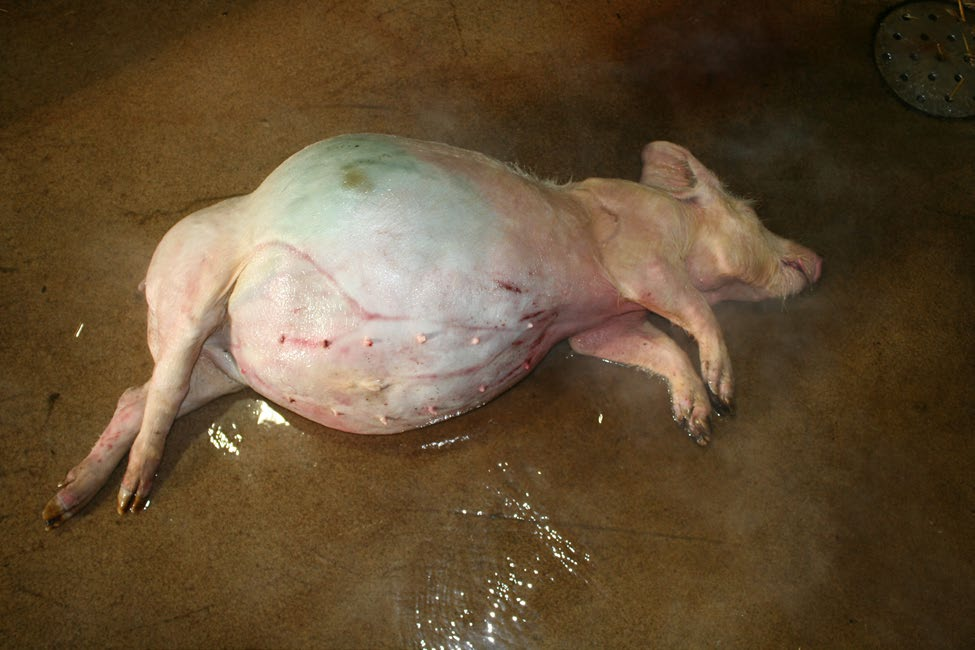
Fattening pig carcass with extensive abdominal extension and emaciation

**Fig. 19:**
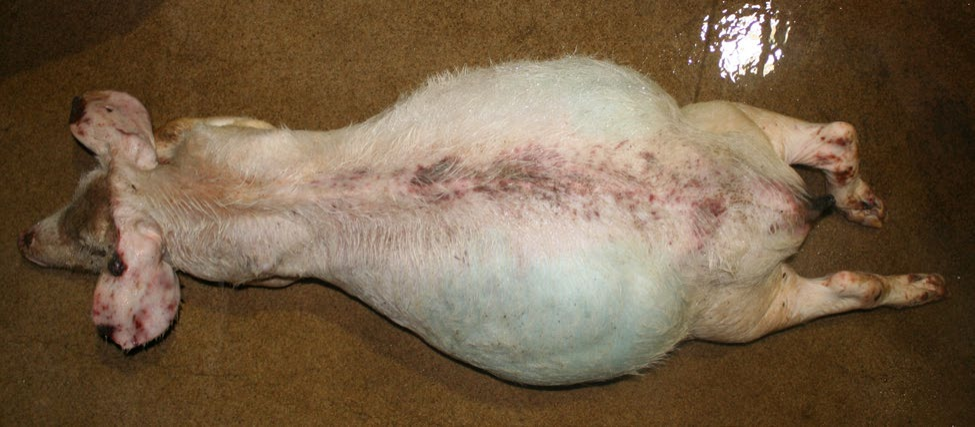
Fattening pig carcass with extensive abdominal extension, emaciation, ulcerative dermatitis and tail necrosis

Extensive distension of the abdomen, indicative of rectal stricture, was observed in 12 carcasses of the German study, whereas this condition was not considered in the Austrian study. All affected carcasses showed additional signs of substantial weight loss or emaciation (Figs. 18 and 19).

### Artefacts and autolysis

**Fig. 20:**
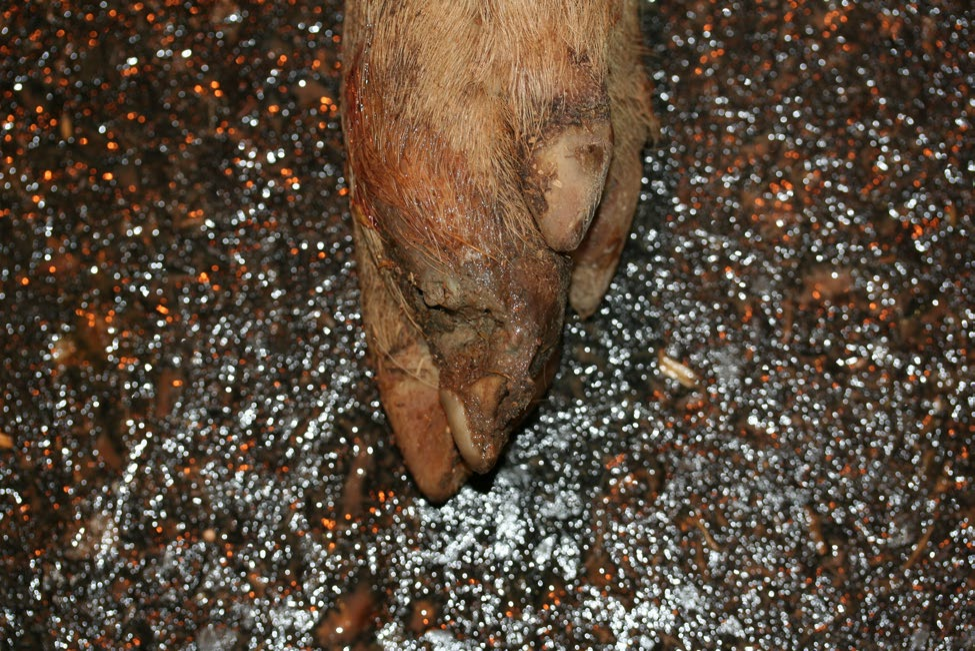
Claw lesion, presumably due to *post mortem* biting damages

**Fig. 21:**
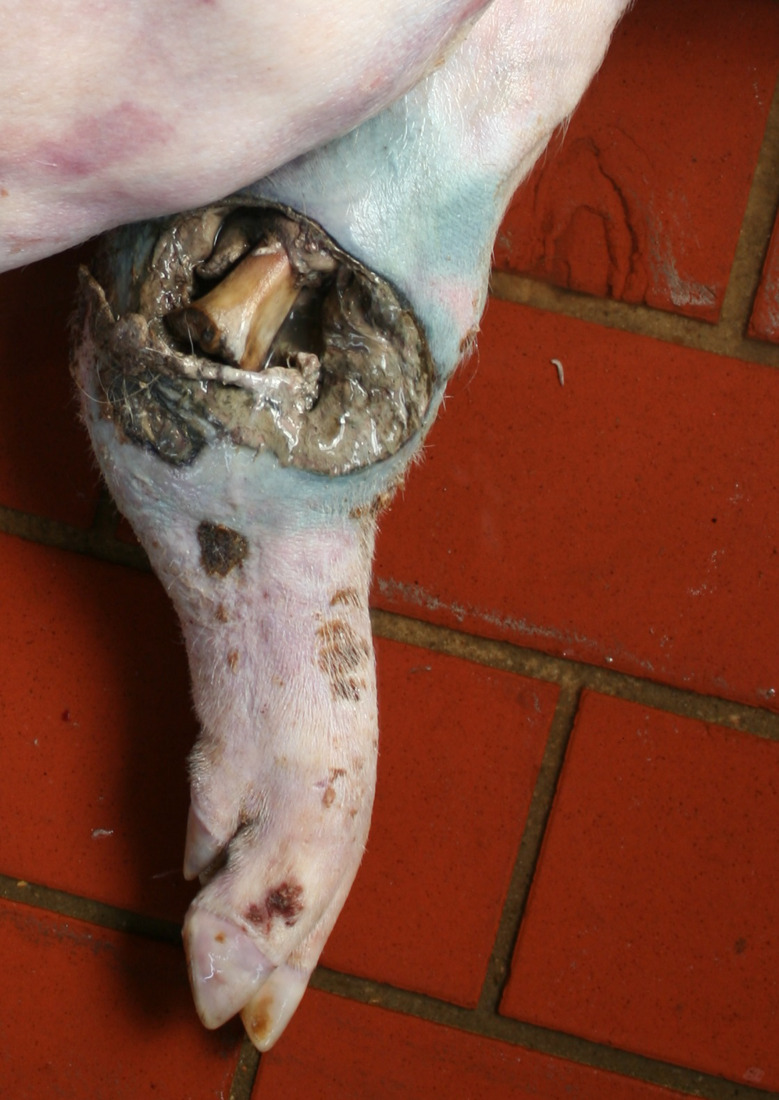
Extensive tissue necrosis in the area of the tarsal joint with exposed distal part of the *os tibia*; exposure of the bone due to *post mortem* tissue loss cannot be ruled out

Disease and injury related findings must always be distinguished from artefacts in the differential diagnosis. Artefacts may be caused, for example, by other pigs in the form of *post mortem* biting damage (Fig. 20) or by the transport of the carcass on the farm or to the rendering plant. In the case of extensive necrotic tissue, it must also be considered that tissue may have been lost during transport; for example, in the pig shown in Fig. 21, it was not possible to determine with certainty whether the bone (*os tibia*) had been exposed during the animal’s lifetime or only exposed due to the *post mortem* loss of the necrotic tissue.

**Fig. 22:**
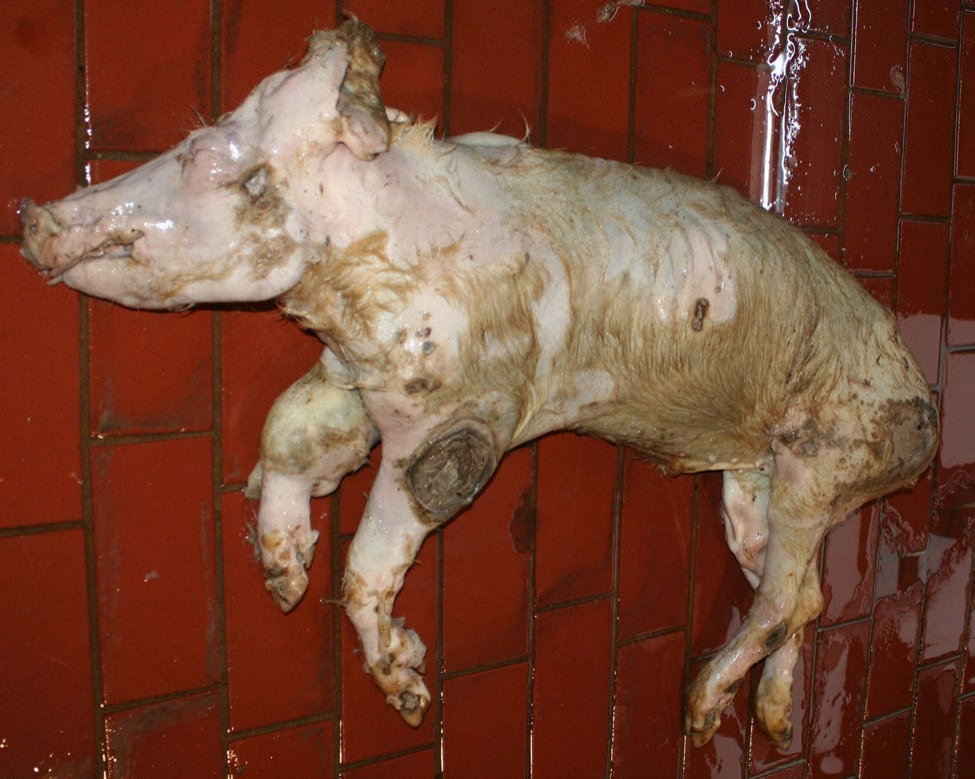
Carcass with high degree of autolysis, pronounced emaciation, skin ulcers and enlargement in the area of the right front carpal joint

As fallen animals were apparently not always reported by the farmer to the rendering plant for collection immediately after death, signs of autolysis were found in some of the animals. However, ulcerative skin lesions, marked emaciation or enlargement of joints were clearly visible even in autolysed carcasses (Fig. 22).

## Discussion

### Perspective and limitations of the study

One question raised by the studies presented was whether substantial pain and/or suffer could be identified by an external examination in pig carcasses delivered to rendering plants. The focus was on findings that were expected to be identified and assessed by experienced farmers or caretakers. However, it should be considered that pain or suffering not associated with externally visible lesions, like gastro-enteric (pain) and respiratory disorders (suffering) [9] –may easily remain undetected. In this sense, the monitoring of carcasses in rendering plants should be understood as a tool to identify the ‘tip of the iceberg” that needs to be followed up by on-farm examinations. On the other hand, the results of *post mortem* examinations may lead to a higher number of animals being recorded as ‘positive’ welfare cases compared to *intra vitam* welfare assessments, as the history (e.g. if a treatment has been given) is usually unknown and needs to be clarified later.

Examination conditions at rendering plants can be challenging. Depending on the individual location, the time available to examine a single carcass may be limited. Several delivery trucks may arrive at the same time, delivering piles of fallen animals that must first be disentangled and placed individually on the floor for assessment is sometimes creating space problems.

### Prevalences

#### Overall prevalence of welfare related findings

Welfare related findings associated with substantial pain and/or suffering were found in 20.8% of all examined pig carcasses in the Austrian study and in 16.0% in the German study. This discrepancy can be explained by the fact that in Austria all animals with at least one pathological lesion, regardless of severity, were classified as having ‘findings potentially associated with substantial pain and/or suffering’, whereas in the German study only pigs with moderate and severe lesions were classified in this way. It should also be noted that the prevalences give the apparent prevalence, whereas the true prevalence could not be calculated because a reliable estimate of the specificity and sensitivity of external inspection of carcasses is not available. The high numbers of affected pigs was confirmed in another study carried out in rendering plants in Germany, in which welfare related lesions were identified in nearly 50% of pigs (*n* = 416) and also cattle (*n* = 420) [5]. However, the higher prevalence of welfare related findings in this study needs to be interpreted in the light of the circumstance that welfare related lesions were recorded regardless the severity. Furthermore, results of official on-farm controls in North-Rhine Westfalia revealed welfare related deficits in 59% out of 387 fattening herds [3].

### Emaciation

Emaciation was a common finding in a total of 6 carcasses in the Austrian study and 215 carcasses in the German study. Emaciation was associated with other findings such as ulcerative skin lesions and tail/ear biting lesions. Emaciation was mainly diagnosed on the basis of clear visibility of the large bones (shoulder, pelvis, ribs, spinous processes) and muscle atrophy, particularly of the longissimus dorsi muscle and the long ischial muscles. These findings are consistent with the assessment of [12], who describe a body weight of ≤ 60% of normal weight as emaciation. The potential link between emaciation and substantial suffering is based on studies focusing on porcine periweaning failure-to-thrive syndrome (PFTS), which examined the consequences of substantial emaciation on the body [13–20]. In addition to the studies in pigs, some studies in humans with anorexia nervosa were also considered [21, 22]. Although the causes of anorexia nervosa in humans are likely to be completely different from the causes of emaciation in pigs, it can be assumed that the consequences of emaciation for the mammalian organism are comparable - especially if both species are monogastric. Typical clinical findings associated with emaciation include anorexia with hollow abdomens, lethargy, growth retardation, muscle degradation, progressive weakness, long hair and dehydration [14, 23]. Pathological findings include superficial gastritis, villous atrophy of the small intestine, superficial colitis and thymic atrophy [15, 17, 19, 23]. Examination of blood parameters showed increased levels of haemoglobin and haematocrit while the levels of sodium, phosphorus and glucose levels were reduced. Changes in these parameters may indicate a disturbance in homeostasis [13]. In human patients with anorexia nervosa symptoms including hypothermia, bradycardia, anaemia, hypokalaemia, hyponatraemia, hypomagnesaemia, hypocalcaemia, hypophosphataemia and hypoglycaemia have been reported [22]. The data show that emaciation, including its final manifestation, is not an otherwise asymptomatic condition. The consequences of emaciation are by no means limited to a ‘slim physiognomy’, but are accompanied by various comorbidities. Particular attention should to be paid to the symptoms which, in addition to the substantial loss of the muscular strength, accelerate the ‘general weakening’ of the animals.

### Swellings

Enlargements were found in 30 carcasses in the Austrian study and in 169 carcasses in the area of joints in the German study. Purulent arthritis was confirmed through incisions in all pigs belonging to a subsample examined in this way. In this context, it should be taken into account that arthritis not associated with visible joint enlargement, non-infectious arthritis and arthrosis are very likely to be missed during external examination of a pig carcass. The selection criterion, the substantial enlargement of at least one joint is known to be clearly associated with severe lameness. Lameness is a term that encompasses a number of deviating findings, including asymmetric weight distribution, a steep gait, increased stride frequency and shortened stride lengths. It also refers to rapid changes between loading and unloading of a limb, known as ‘tapping.’ In addition, affected pigs often exhibit an arched spine [8, 24]. In these actions lame pigs reduce weight bearing to avoid or at least reduce pain [8]. Lameness is also associated with behavioural changes such as self-separation by lying down along walls and reducing interactions with other pigs in the group [25–27]. Over all, lameness is assessed as a clear sign of pain [8, 26, 28–30].

### Foot lesions

Foot lesions, including claw, dewclaw and coronary band alterations, were found in 23 carcasses in the Austrian study and 78 carcasses in the German study. The lesions were mostly found in breeding pig carcasses. As locomotion cannot be assessed in dead pigs, only very pronounced lesions which are known to always cause significant lameness [31], were assessed as being substantially painful. These lesions were complete loss of a horn shoe, amputation of a dewclaw and deep coronary band lesions. Substantial overgrowth of the claws was assessed potentially causing suffering. As locomotion could not be assessed, only claw formations clearly incompatible with normal locomotion were scored causing suffering. The assessment took into account that claw overgrowth usually affects all legs. Considerably overgrown claws have a substantial impact on the welfare of pigs as they restrict movement and make it impossible to maintain a physiological standing position. The consequences are a reduction in time spent standing and eating and an abnormal lying down behaviour [32–34].

### Ulcerative skin lesions

Perforating ulcerative skin lesions, affecting the subcutis or even the underlying bone were found in 67 (Austria) and 82 Germany) carcasses. A further 114 carcasses showed ulcerative lesions confined to the superficial layers of the skin. The distinction between superficial and deep lesions was made on the basis of a score evaluated for the grading of shoulder ulcers [35, 44]. This score, developed for histopathology, has now been applied to clinical findings, showing that it is generally possible to distinguish superficial from deep ulcerative lesions in a clinical examination [36]. Ulcerative skin lesions can be assessed to cause substantial pain when all skin layers (dermis, epidermis, subcutis) or even the underlying tissues are involved. Studies in pigs evaluating pain induced by ulcers mainly refer to shoulder ulcers in sows [37–41]. Behavioural changes such as reduced lying time, increased frequency of postural changes, increased standing and reduced nursing frequency have already been observed in sows with shoulder ulcers of moderate size (3 cm in diameter) [39]. Another behavioural change can be increased rubbing against fixtures of the farrowing crate or can be induced by palpation [37, 39]. In addition, a correlation has been found between pain-associated responses to palpation of shoulder ulcers and the depth of the lesion [38]. In humans, people describe their perception of pain associated with pressure ulcers of class 2 to 4 from ‘discomforting’ to ‘distressing’ to ‘horrible’ [42]. As the nociceptive systems of humans and pigs have comparable traits [28], and as histological examination of shoulder ulcers reveals inflammatory reactions and traumatic neuromas [37], it is reasonable to assume that ulcerative skin lesions cause pain in pigs [43]. Depending on the size and depth of the lesions substantial pain might affect the pig.

### Biting lesions

Biting lesions were found in 103 carcasses of the Austrian study. In the German study, 206 carcasses showed tail biting and in 118 carcasses ear biting lesions were seen. In the German study, the lesions were characterised by loss of substance combined with signs of inflammation and necrosis in most carcasses. In almost all carcasses with tail biting lesions it was very likely that the pigs had got their tails docked within a few days after birth, as tail docking is a routine management procedure in conventional pig production in Austria and Germany. Therefore, it can be assumed that most of the biting lesions were found in pigs that got their tails previously docked. An accurate estimate is not possible as the loss of substance due to biting makes it impossible to differentiate between pigs with docked and non-docked tails. In the German study, tail and ear lesions were scored using the criteria ‘inflammation’, ‘necrosis’, and ‘loss of substance’. Loss of substance ranged from localised tissue loss to loss of the entire tail, sometimes including injury of the tail root and the surrounding tissues. It was also determined whether the injury actually occurred while the pig was alive, as bite wounds can develop after a pig has died. Tail biting and resulting lesions are generally associated with pain in pigs [45-47]. Hanging or tucked tail postures are indicative of inflamed wounds [48, 49]. Although ear biting lesions of a certain degree are likely painful for the affected pigs, this issue is not discussed in related papers. In this study, tail or ear lesions with extensive necrosis and/or purulent inflammation were evaluated as potentially painful. Tail biting lesions are associated with secondary systemic infections leading to arthritis or abscess formation in the spine or lung [45-47, 50, 51]. In the German study, tail biting lesion were positively associated with arthritis and ear biting lesions.

### Umbilical outpouchings with large size and/or ulcerative skin lesions

In the German study, large sized and/or injured umbilical outpouchings were found in 28 carcasses. Umbilical hernia, enterocystoma and herniated enterocystoma are clinically evident as umbilical outpouching [52]. Umbilical outpouchings are common in pigs and may affect their welfare [53]. The case fatality is usually high [54]. Apart from smaller outpouchings with intact skin, which may not or only slightly affect welfare, larger outpouchings with impaired reducibility or skin wounds are considered relevant in terms of pain and suffering. The Danish Veterinary Health Council considered the euthanasia of an affected pig to be ‘timely” when the size of an umbilical outpouching becomes large (6–10 cm in 15 to 35 kg pigs or > 15 cm in finisher pig) or when movement is obviously impaired or when the skin is ulcerated [55]. In this study, in 13 carcasses the umbilical outpouchings, which clearly exceeded the size of 15 cm and/or showed extensive or deep ulcerative lesions, were evaluated having caused substantial pain and/or suffering.

It is uncertain whether umbilical outpouchings without intestinal incarceration or adhesions, or ulcerative skin lesions are painful for the pig [8]. However, intra-abdominal lesions were frequently found in a study on pigs with wounded umbilical outpouchings [52]. Reduced lying time in pigs with umbilical outpouchings (after transport) [56] and their reduced willingness to engage in locomotor and social activities [53] may indicate pain and suffering. Reduced lying time may be a consequence of impaired lying in sternal recumbency, which is the predominant lying position in pigs. Ulcerative skin lesions are considered to be painful [57]. Therefore, it can be expected that ulcerative lesions on hernias are also painful. Ulcerations are the most common complication leading to euthanasia in pigs affected by umbilical outpouchings [54]. The risk of skin ulceration is positively associated with the size of the hernia and is increased in hernias where the content cannot be replaced into the abdomen [54].

### Rectal stricture

Extensive abdominal distension, indicative for rectal stricture, was found in 12 carcasses in the German study. Rectal stricture is a frequently occurring consequence of an injured rectal prolapse or an ulcerative proctitis caused by bacterial infection [58]. The stricture is caused by an annular cicatrisation of the rectal wall, that develops a few centimetres anterior to the anorectal junction [59]. Affected pigs develop emaciation, colonic dilatation (megacolon) and compression atrophy of the abdominal and thoracic viscera due to the progressive obstruction [58, 59]. The initial lesion usually develops 4 to 8 weeks before the onset of stricture [59]. Although pigs that develop clinical signs of a rectal stricture are known from practice to show substantial signs of suffering, no studies have been published addressing pain or suffering caused by this lesion. In the present study, the substantial suffering in pigs with rectal stricture was identified on the basis of the emaciation seen in all affected pigs.

### Assessments at rendering plants as a tool to monitor animal welfare - consequences and recommendations

The results of the present study suggest that, in both Germany and Austria, a subset of pig farming operations systematically fails to provide adequate care for severely diseased and/or injured animals. The frequency and severity of these deficiencies indicate that they cannot be dismissed as isolated incidents or attributed solely to a few non-compliant individuals (‘black sheep’). External examination of pig carcasses at rendering plants may offer a basis for identifying potential violations of § 17 (2b) of the German Animal Welfare Act [59] and § 5 (1) of the Austrian Animal Welfare Act (60).

Pig farmers are responsible not only for the herd as a whole but also for the welfare of each individual animal [60]. Animals, dependent on regular human care must be visually inspected at least once daily to ensure timely identification of health issues [60, 61]. This requirement is grounded not only in animal welfare and legal obligations but also aligns with the farmer’s economic interests, as early detection and treatment of illness or injury can significantly improve recovery outcomes [62]. If clinical signs of disease or injury are observed, appropriate measures must be initiated without delay, including veterinary consultation where necessary [60, 61]. Affected animals must be housed appropriately and, if needed, separated from the group to prevent further harm or stress. In accordance with Council Directive 98/58/EC [63], which prohibits causing animals unnecessary pain, suffering, or injury, animals for which treatment is deemed impossible or disproportionate must be euthanised without delay.

### Steps towards the implementation of a uniform assessment scheme to be used at rendering plants

The current carcass disposal procedure does not include routine inspection of fallen animals for animals for welfare-related lesions. *Post mortem* inspections are only carried out sporadically on the initiative of dedicated official veterinarians [4]. In order to increase the detection rate of carcasses with lesions relevant for animal welfare, it is necessary to establish a standardised and feasible protocol that can be routinely applied in all rendering plants. Magenschab [64] and Haas [65] presented a concept in which truck drivers are trained to recognise signs associated with substantial pain and suffering in the fallen animals on the basis of defined cardinal symptoms when picking them up. Conspicuous animals should be marked with an additional ear tag. At the rendering plant, the pre-selected carcasses would be set aside for a final inspection by official veterinarians based on a standardized inspection protocol. Haas (66) demonstrated that the trained truck drivers did not overlook any animal welfare-relevant cases and only gave false positive assessments for single carcasses. This concept could in principle be integrated into the routine operations of animal carcass processing plants with little additional effort. At the same time, it would significantly improve the accuracy of official veterinary animal welfare controls. However, traceability is only possible if the fallen animals are provided with an official identification (ear tag or tattoo) or if the carcasses are collected individually from a farm. For countries with large herds, such as Germany, we recommend that all livestock owners be required to mark each carcass with an ear tag, containing the farmer’s identification number. This number would make it possible to identify the person responsible for the animal at the time of death. As piglets normally have an ear tag allowing the identification of the owner, additional tags have only been applied to those dead pigs where the owner is no longer the piglet producer. As each dead pig must be removed from the pen ‘by hand’ it seems practical and reasonable to mark the carcass with an additional plastic ear tag [11].

## Conclusions

Both studies included in this paper show that a noteworthy number of pig carcasses with animal welfare-relevant lesions were delivered to Austrian and German rendering plants. The results of the external examination of these carcasses indicate that the affected animals experienced considerable pain and suffering until their death. It can also be concluded these diseased and injured animals were not euthanised in a timely manner. Routine external inspection of carcasses delivered to rendering plants according to a standardised examination protocol and the resulting interventions could contribute to improve the handling of diseased and injured animals. In particular, the timely euthanasia of pigs after unsuccessful treatment could prevent animal pain and suffering. In addition, the inspection of carcasses in rendering plants could help to better identify farms at high risk of significant animal welfare problems.

## Data Availability

Please contact the corresponding author for data requests.
